# Effect of the Irrigation Agitation Technique on Postoperative Pain in Teeth With Symptomatic Irreversible Pulpitis

**DOI:** 10.7759/cureus.61331

**Published:** 2024-05-29

**Authors:** Beenish Abbas, Emaan Ahsin, Summiya Asghar, Faiza Habib, Hina Ishaq, Nasar Um Min Allah

**Affiliations:** 1 Pediatric Dentistry, Foundation University Islamabad, Islamabad, PAK; 2 Periodontics, Foundation University Islamabad, Islamabad, PAK

**Keywords:** visual analog scale, postoperative pain, manual dynamic agitation, symptomatic irreversible pulpitis, endodontic irrigation, endodontic treatment

## Abstract

Introduction

Irrigation of the root canal system is a vital step in endodontic treatment aimed at disinfecting the canal. The efficacy of irrigation can be improved by various irrigation agitation methods. One such novel method of interest is the manual dynamic agitation (MDA) technique. However, the effect of MDA on postoperative pain as compared to needle irrigation (NI) with sodium hypochlorite has been scarcely explored. This study aimed to compare the effects of NI and MDA techniques on postoperative pain in teeth with symptomatic irreversible pulpitis.

Materials and methods

This quasi-experimental study was conducted at the Department of Operative and Paediatric Dentistry, Fauji Foundation Dental Hospital, over four months after gaining ethical approval. One hundred and sixty-eight participants diagnosed with symptomatic irreversible pulpitis were enrolled in the study through the purposive sampling technique. The participants were divided into two groups based on the irrigation technique used: Group A (NI) and Group B (MDA). Postoperative pain was recorded after six hours, 24 hours, 48 hours, and seven days using the 0-100mm visual analog scale (VAS). The VAS scores were compared using an independent sample t-test.

Results

Out of 168 participants, 48.2% were in Group A and 51.2% in Group B. The study found that VAS pain scores for Group B (MDA) were significantly lower at six hours, 24 hours, 48 hours, and seven days as compared to Group A (NI), with a *p*-value less than 0.001.

Conclusion

This study shows that the MDA technique leads to decreased postoperative pain both immediately after endodontic treatment and a week later as compared to the NI technique. Hence, the use of MDA can aid in controlling postendodontic pain and, therefore, ensure smoother recovery and increased patient satisfaction.

## Introduction

The root canal system is anatomically complex. Hence, 35% of the root canal system remains untouched after mechanical instrumentation [1], leaving intracanal and extraradicular biofilm harboring microorganisms including Enterococcus faecalis and yeast, mainly Candida albicans, which adversely affects the outcome of endodontic treatment [2]. The aim of using an irrigation solution (sodium hypochlorite and saline) is to inhibit microbial growth, dissolve the smear layer, and remove pulp tissue. Irrigation is an essential part of endodontic treatment for effective disinfection [3]. The effectiveness of irrigation is affected by the complex morphological features of the apical area, making complete disinfection and the elimination of resistant root canal microbiota challenging. This difficulty is compounded by inadequate flushing of irrigants [4]. Pulpal and periapical diseases have primarily bacterial etiologies, and the purpose of chemo-mechanical debridement is a major reduction in the load of intracanal bacterial species by making use of antibacterial irrigants and enhancing irrigant delivery to the morphological intricacies of root canal space to ensure peri-radicular tissue healing [5].

Owing to the morphological complexities of the root canal system, a syringe and needle alone are insufficient to eradicate resistant bacteria like Enterococcus faecalis to a level compatible with peri-radicular tissue healing. Irrigation can be improved via various irrigation agitation methods. Agitation of irrigants can be achieved by manual agitation of fluid by filing motion using small stirring movements or automated methods like sonic and ultrasonic instruments. In the passive ultrasonic irrigation technique, a smooth wire or passive endodontic file is activated by ultrasonic energy, causing cavitation and acoustic streaming in the fluid present within the confines of the canal space and generating hydrodynamic shear forces for adequate debridement of the root canal [6]. Sodium hypochlorite at a concentration of 5.25% shows antibacterial and tissue dissolution properties; EDTA with the concentration of 18% helps in the removal of the smear layer; alternate use of sodium hypochlorite and EDTA is still ineffective in rendering infected root canals free from cultivable bacteria [7]. This highlights the need for more effective strategies. Some of these novel techniques are photon-induced photo-acoustic streaming (PIPS) and manual dynamic agitation (MDA).

PIPS is described as laser-activated irrigation using an erbium laser (2940 nm) in a pulse repetition mode to activate irrigation flow through the morphological intricacies of the root canal system [8]. MDA involves the repeated insertion of a well-fitting gutta-percha cone at an accurate working length after mechanical instrumentation of the canal; this activates irrigation fluid-generating hydrodynamic shear forces resulting in biofilm removal and is effective in rendering canals free from cultivable bacterial species since it deals with the pattern of irrigant flow, penetration, exchange and the forces produced within the root canal space [9]. Various preoperative and intraoperative factors increase the occurrence of postoperative pain (PP) after endodontic treatment. Some of the etiological factors known to have an increased association with PP are the presence of preoperative periapical disease, a history of preoperative pain, debris, and irrigant extrusion in the periapical area during endodontic treatment [10]. Another etiological factor associated with PP is that sodium hypochlorite irritates periapical tissues; however, the antibacterial and tissue dissolution properties of sodium hypochlorite are well known. Thus, there is a search for root canal irrigants with antimicrobial properties but less potential to induce periapical tissue irritation. One such promising agent is chlorhexidine gluconate, a 0.12% solution, which is effective in the chemical control of biofilm but has less potential to cause PP [11]. This study aimed to evaluate the effect of the needle irrigation (NI) technique (no agitation) and MDA techniques on PP. Literature is scarce on the comparative evaluation of irrigation agitation techniques on PP after endodontic treatment in molar teeth with symptomatic irreversible pulpitis. However, the effects of other variables like the number of appointments, intracanal medicaments, and canal preparation techniques on PP have been evaluated in various studies.

## Materials and methods

This study prioritized participant anonymity, privacy, and informed consent while closely adhering to ethical guidelines. The study was approved by the Ethical Review Board (ERB) of the Foundation University College of Dentistry & Hospital, Foundation University Islamabad, with ERB Approval Number: ERC # FF/FUCD/632.

In this study, 168 study participants were recruited after applying inclusion and exclusion criteria to the pool of patients reporting to an outpatient restorative and endodontic clinic. Study participants were enrolled for a period of four months, from September 2021 to June 2022.

The inclusion criteria encompassed patients of both genders in the age range of 18 to 60 years. They had ASA I and II status with no significant systemic disability. It also included patients with their maxillary and mandibular premolar and molar teeth diagnosed with irreversible pulpitis, or acute irreversible pulpitis restorable through endodontic treatment. Both molar and premolar teeth were included as recent studies indicate that postendodontic pain does not exhibit any significant correlation with the number of roots of the selected tooth [12]. Both maxillary and mandibular teeth were included in the study as according to the recent literature the type of tooth (maxillary and mandibular) had no significant effect on postendodontic pain [13]. The tooth under treatment must have an antagonist tooth present with good occlusal contact. Patients with a preoperative pain score in the moderate to severe category (45-100 mm) score on a visual analog scale (VAS) of 0-100 mm [14]. The exclusion criteria applied to participants were patients with severe renal disease, bleeding disorder, or immunocompromised status; lactating and pregnant patients; teeth showing radiographic signs of internal and external resorption; cases requiring retreatment due to endodontic failure; teeth with immature open apex; teeth with calcified canals; necrotic with chronic periapical abscess/Phoenix abscess(due to the potential bias); patients who have taken narcotic, analgesic, or anti-inflammatory drugs within the last 24 hours; and patients showing malocclusion.

A detailed intraoral and extraoral examination was done for each patient. A clear diagnosis of symptomatic irreversible pulpitis or acute periapical abscess was established based on history, clinical findings, and investigations. Periapical radiographs were also taken for each patient to examine the periapical status of each tooth under treatment. Several factors can significantly influence postendodontic pain, including the presence of pulp tissue remnants, inaccuracies in working lengths, and over instrumentation according to recent research [15]. These factors were accounted for when formulating inclusion and exclusion criteria. Verbal and written informed consent was obtained, with the right to withdraw from the study at any point in time.

Simple randomization via the lottery method was employed to allocate participants to two groups, i.e., the control group (Group A) for the NI technique and the treatment group (Group B) for the MDA technique. The treating clinician was not blinded to the treatment group due to the nature of the intervention, but patients were blinded to the procedure. A specialist endodontist performed endodontic treatment under rubber dam isolation using a high-speed contra-angled handpiece under copious water-cooling. The access cavity was made followed by establishing the working length using an electronic apex locator Dentaport ZX^TM®^ (J. Morita Co., Kyoto, Japan) and an intraoral periapical radiograph. The master apical file employed exceeded the initial apical file by three sizes as recommended by the literature. This enlargement causes the prolonged presence of irrigants within the canals, enhancing bacterial removal [16].

Thorough mechanical instrumentation and copious irrigation using 3% sodium hypochlorite (NaOCL) using a syringe kept 2mm short of the working length were done. The needle used for both groups was a 30-gauge side-vent needle. However, the final irrigation protocol was divided into two groups. In Group A, the control group, canals were irrigated with 5 ml of NaOCl per canal with a needle using no activation protocol. In Group B (MDA), each canal was flooded with an irrigating solution and activated using a manual dynamic agitation technique. The gutta-percha cone (Gapadent, Tian Jin, China) corresponding to the master apical size was activated at a frequency of 100 push-pull strokes per minute. Patients were dispensed with clear postoperative instructions, and analgesics prescribed were ibuprofen 400mg and paracetamol 500 mg, according to the clock medication regime (ibuprofen 400 - 800mg TDS and/or 500mg acetaminophen QID). PP was recorded at six hours, 24 hours, 48 hours, and seventh day of endodontic treatment using the VAS pain score, classified as 0-4 mm as no pain, 5-44 mm as mild pain, 45-74 mm as moderate pain, and 75-100 mm as severe pain [17].

The data was entered into the data management software and statistical analysis was done using IBM SPSS Statistics for Windows, Version 23 (Released 2015; IBM Corp., Armonk, New York, United States). The descriptive statistics were reported as mean values for continuous variables including age and VAS scores along with standard deviations, whereas frequency and percentages were reported for categorical variables. Inferential statistics included VAS score comparison between groups using an independent sample *t*-test or a one-way ANOVA test depending on the number of groups. The *p*-values of <0.05 were considered significant. 

## Results

There were 168 patients enrolled in the study. The majority of the participants were females, 152 (90.5%), while 16 (9.5%) were males. The participants underwent root canal procedures and were divided into two groups based on the technique involved. There were 81 (48.2%) participants randomized to NI technique Group A, whereas 86 (51.2%) participants were randomized to MDA technique Group B. The overall mean age was 37.93 ± 11.7 years. The demographic characteristics of the two study groups are summarized in Table 1.

**Table 1:** Baseline characteristics of participants belonging to Group A and Group B (n=168) Group A: Needle irrigation technique Group B: Manual dynamic agitation technique

Characteristics		Frequency n (%)		p-value
		Group A	Group B	
		(n=81)	(n=86)	
Gender	Male	10 (12.3%)	5 (5.8%)	0.140
	Female	71 (87.7%)	81 (94.2%)	
Mean age (years)		37.48±10.5	38.58±12.7	0.548

The pain scores were assessed for each participant using the VAS, with values ranging from 0 to 100 mm at six hours, 24 hours, 48 hours, and seven days postendodontic procedure. The VAS pain scores were compared between two study groups using an independent sample t-test. A significant difference in the VAS pain score was found at six hours, 24 hours, 48 hours, and seven days post-treatment between the NI technique and MDA technique groups, as shown in Table 2.

**Table 2:** Comparison of the mean VAS pain score at six hours, 24 hours, 48 hours, and seven days post-RCT procedure between Group A and Group B Group A: Needle irrigation technique Group B: Manual dynamic agitation technique VAS: Visual analog scale; RCT: root canal treatment

Post-procedural follow-up time	Mean VAS pain score		p-value
	Group A (n=81)	Group B (n=86)	
At six hours	45.63±25.4	12.16±17.4	<0.001
At 24 hours	39.14±9.13	9.13±19.6	<0.001
At 48 hours	23.17±21.32	6.45±16.4	<0.001
At seven days	10.74±19.1	0.93±8.6	<0.001

**Figure 1:**
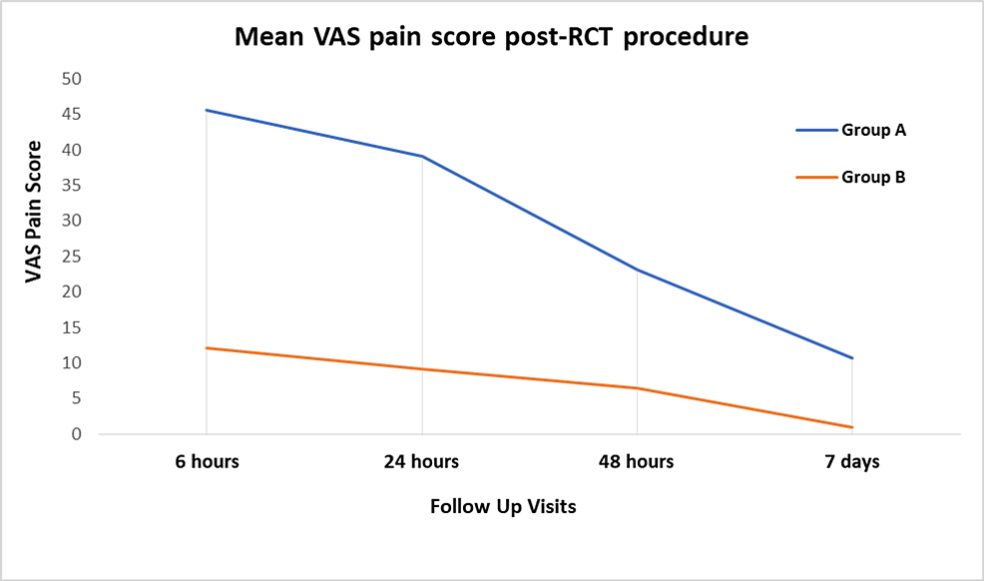
Comparison of the VAS pain score between Group A and B at post-procedural follow-up visits Group A: Needle irrigation technique Group B: Manual dynamic agitation technique RCT: root canal treatment; VAS: visual analog scale

At six hours post-procedure, the mean VAS pain score was significantly lower in the MDA technique group as compared to the NI technique group, i.e., 12.16±17.4 vs. 45.63±25.4 (*p *< 0.001). Similarly, at 24 hours, the mean VAS pain score was significantly lower in the MDA group with a mean value of 9.13±19.6 as compared to the NI technique group with a mean value of 39.14±9.13 (*p *< 0.001). At 48 hours, a similar trend of significantly lower mean VAS pain score was observed for the manual dynamic agitation technique group as compared to the NI technique group, i.e., 6.45±16.4 vs. 23.17±21.32 (*p *< 0.001). At seven days postendodontic procedure follow-up, again, the mean VAS score was significantly lower for patients belonging to the MDA group with a mean value of 0.93±8.6 as compared to the NI technique group with a mean of 10.74±19.1 (*p *< 0.001). The graphical trend of mean VAS pain scores is given in Figure 1.

## Discussion

Root canal treatment is a routine dental procedure performed by general dentists and endodontists alike. Unfortunately, patients often experience varying levels of pain after the procedure [18]. The objective of this study was to compare PP after root canal treatment using two irrigation methods on teeth with symptomatic irreversible pulpitis. These two methods were the NI technique and the MDA technique. A strength of this study is that it explores and aids in bridging the gap in knowledge about MDA and PP, which is an understudied area of research. As pain is subjective to the patient, it may prove to be challenging to evaluate. Therefore, the method of assessment or questionnaire being used should be well explained to the patients [19]. For this purpose, the VAS pain score was used, which is validated, reliable, and easy to understand [14]. The findings of the current study show that there is significantly less PP with the use of MDA at six hours, 24 hours, 48 hours, and seven days. This is also in agreement with the general trend that PP steadily decreases over seven days, regardless of the irrigation technique used [20].

In contemporary endodontics, the literature has established that the use of irrigation activation techniques is more effective in cleaning root canal systems than simply flushing the canal with standard irrigants [21,22]. This has led to an interest in the effect of activation techniques on PP as well. In a systematic review by Susila and colleagues, it was found that most irrigant activation techniques lead to decreased PP, with the exception being MDA. This is in direct contradiction to the findings of the current study. The review also showed that the difference in PP using activation methods and NI was only significantly less in the first 48 hours after treatment with any activation method used [23]. Again, this is different from our findings, which show significantly less pain with MDA at seven days as well. It should be noted that the review found only one study that assessed PP with MDA, and to the best of our knowledge, there has not been any other study [9] that has done so. Hence, the scarcity of literature could explain why the findings in the present study differ from those in the review. In the study conducted by Topçuoğlu et al., PP was compared with the use of MDA, passive ultrasonic irrigation, NI, and sonic agitation [9]. The findings showed that MDA had the highest PP; however, this was only true for the first 24 hours, after which the differences in PP were not significant across techniques, including MDA. Like the present study, Topçuoğlu et al. also utilized VAS to quantify pain. However, unlike this study, they also used 17% EDTA in the canals along with 3% NaOCl [9]. Furthermore, their sample size for evaluating PP with the use of MDA was smaller than in this study, which could be a probable reason for the contrasting results.

The aims of cleaning the root canal system are to reduce levels of microbes (primarily gram-negative anaerobes Enterococcus faecalis), dissolve pulp tissue, disrupt biofilm, and remove the smear layer and debris [22]. Failure to clean the canals sufficiently can lead to a poor prognosis and incomplete treatment which can cause postoperative pain and flare-ups [24,25]. NI alone is not the most effective method of cleaning the canal due to the complexities of the root canal systems [22]. Moreover, NI is only capable of delivering irrigant up to 0-1.1mm beyond its tip [26]. Studies have shown that the MDA technique leads to more effective removal of debris and better dentin penetration which results in improved cleanliness of the canals [21,27]. This could be a possible cause of decreased PP levels in our study. Another common etiology of PP is irrigant extrusion into periapical tissue [28]. NaOCl is corrosive and causes tissue necrosis; hence, extrusion of irrigant beyond apical constriction will damage periapical tissue and cause pain. The irrigation technique used affects the amount of apical extrusion of irrigant [29] with all activated techniques showing significantly more extrusion than nonactivated methods of irrigation [26]. Despite this, NI has been shown to result in higher levels of PP as compared to activation methods [23].

One potential limitation of this study is the use of a quasi-experimental design. Unlike randomized controlled clinical trials, quasi-experimental designs do not involve randomly assigning participants to experimental and control groups. Moreover, the present study could not be double-blinded since knowledge of the operator is crucial to carrying out the clinical procedure. The study aimed at the targeted selection of participants who meet specific criteria relevant to the study's objectives and a purposive sampling technique was used. To reduce the selection bias between the groups that could influence the outcomes, we employed simple randomization via the lottery method and matching techniques to pair the participants in control and experimental groups. To further mitigate this, we used purposive sampling to ensure that the experimental and control groups were comparable in terms of key characteristics which enhances the relevance and depth of the data collected. Besides, the study included relevant covariates in the statistical analyses to adjust for potential confounders and better isolate the effect of the intervention. Furthermore, the study provided a detailed description of the context, participant characteristics, and intervention to help readers understand the specific conditions under which the findings were obtained. Another possible limitation was the subjective nature of PP which depends on the cultural, individual, and economic background of the participants. Therefore, we used the VAS pain score to rate the pain severity since this scale is considered a reliable and valid tool for the evaluation of pain relief [14]. All the technique and operator-related variables were minimized and controlled by allowing one single operator to perform all the root canal therapy procedures [30]. With scarce literature exploring the effect of MDA on PP, future research employing randomized controlled trials with more standardization involving single-rooted or multi-rooted teeth would be beneficial to increase its validity, reliability, and generalizability.

## Conclusions

In conclusion, this study demonstrates that using MDA during endodontic procedures significantly reduces postendodontic pain as compared to the NI technique. The enhanced efficiency of irrigant distribution with MDA ensures a more thorough root canal cleansing while also minimizing residual debris, potentially reducing inflammation and subsequent pain. The simplicity of incorporating MDA into existing protocols makes it a compelling option for improving patient comfort and satisfaction without disrupting established dental practices. As advancements are made in the field of endodontics, adopting techniques like MDA holds promise for enhancing patient experiences and improving standards of care. The immediate benefits observed in the present study suggest that MDA merits consideration for routine use.
